# An Analysis of Diagnostic Discrepancies in the Pathology Departments of Wigan and Salford

**DOI:** 10.7759/cureus.99460

**Published:** 2025-12-17

**Authors:** Amira Salem, Muhammad Abdulvahab, Hamza Ahmed, Amal Asar

**Affiliations:** 1 Spinal Surgery, Salford Royal NHS Foundation Trust, Manchester, GBR; 2 Trauma and Orthopaedics, Salford Royal NHS Foundation Trust, Manchester, GBR; 3 Histopathology, Salford Royal NHS Foundation Trust, Manchester, GBR

**Keywords:** anatomical pathology, diagnostic discrepancies, error, histopathology, mdt meetings, surgical pathology

## Abstract

Aim: The aim of this audit is to evaluate diagnostic discrepancies occurring between April 2022 and March 2024 within the Wigan and Salford pathology (PAWS) department. The audit measures the rate and category of errors, identifies the stages at which they occurred, assesses the methods by which they were detected, and considers their impact on patient care, comparing the results to national standards and published data to identify trends and areas of improvement.

Materials and methods: This study was conducted through retrospective analysis of data extracted from the minutes of discrepancy meetings held during the specified two-year period. Each recorded discrepancy was reviewed and categorised according to error type, stage of occurrence, subspecialty and sample type, and method of identification.

Results: This audit found that 56% of the identified discrepancies were of category B (discrepancies in microscopy). Specialties most involved in discrepancies were gastrointestinal tract (GIT) and breast, with biopsy samples being the most specimen type that is prone to error. It was also concluded that the multidisciplinary team (MDT) review was the most effective safety netting method for error identification and rectification at PAWS labs.

Conclusion: The findings highlight the importance of having scheduled time for pre-MDT discussions in an era where histopathology departments are increasingly overstretched. We also highlight the importance of utilising in-lab imaging technologies to better assess post-radiotherapy specimens and biopsies. Strengthening these safety-netting strategies has the potential to reduce diagnostic discrepancies and improve overall patient care.

## Introduction

Errors are inherent to both human activity and healthcare processes. In surgical pathology, errors can occur unavoidably at any stage of laboratory testing--whether in the pre-analytical, analytical, or post-analytical phase. However, the main challenge is the timely identification and rectification of these errors to ensure effective patient safety [1]​. This audit report has been prepared to evaluate the diagnostic discrepancies for the period between April 2022 and March 2024. 

Our goal is to provide an independent and objective evaluation of the department's reported discrepancies during that time frame, identifying both strengths and areas for improvement. This audit outlines our findings, recommendations, and any corrective actions necessary to enhance the department's discrepancy processes' efficiency. 

According to the Royal College of Pathologists: “Laboratories shall ensure that errors, from specimen collection through laboratory processes to receipt of report, including errors of interpretation and clinical advice, are logged, and reviewed systematically, with evidence of effective learning. Laboratories shall be able to demonstrate measures introduced to reduce the likelihood of similar future errors, and that these measures are evaluated for effectiveness” [2].

Locally, several measures are available to report and manage discrepancies within the hospital system. One such measure is DATIX (RLDatix, Chicago, Illinois, USA), a structured process for error logging that is accessible to all hospital departments. It serves as a formal mechanism for documenting and tracking incidents to ensure accountability and transparency. Another option is the corrective and preventive action (CAPA) process, which is primarily used for documentation purposes but does not always require the formal procedures associated with DATIX reporting. Additionally, local discrepancy meetings are held to promote learning and improve decision-making. These meetings are generally informal and do not involve multiple parties, focusing instead on reflection and improvement within local teams [2]. 

Discrepancies are reviewed in these local meetings for several key purposes. Firstly, they aim to establish whether an error has indeed occurred and to categorise it appropriately. Secondly, the review helps determine whether the issue is an isolated incident or part of a broader systemic problem, in which case corrective measures can be discussed and implemented to close any identified gaps. Thirdly, the meetings assess whether patient harm has resulted from the discrepancy and whether formal governance procedures, such as a DATIX report, need to be initiated. Finally, these sessions serve as valuable learning opportunities where staff members of various grades and trainees can learn from others' mistakes, enhancing their understanding and improving their future clinical practice [2]. In this study, we focus on the cases discussed in discrepancy meetings between April 2022 and March 2024.

## Materials and methods

Aim

To review and evaluate diagnostic discrepancies discussed at discrepancy meetings in the Cellular Pathology Departments of Wigan and Salford between April 2022 and March 2024.

Objectives 

The objectives of the review are multifaceted and aim to enhance the understanding and management of diagnostic discrepancies. Firstly, it seeks to identify the proportion of discrepancies across all diagnosed cases each year and to compare these findings against national standards to evaluate performance and consistency. Secondly, it aims to assess the effectiveness of existing safety net measures in detecting discrepancies and preventing any resulting clinical impact, thereby ensuring patient safety. Thirdly, the review will describe how discrepancies are distributed across various subspecialties, which can help identify areas requiring targeted improvement or additional support. Lastly, it will analyse the role of discrepancy meetings in shaping standard operating procedures (SOPs) and contributing to ongoing quality assurance within the department. 

Standards 

The Royal College of Pathologists' datasets and published data regarding diagnostic discrepancies in surgical pathology were used as benchmarks. 

Methods

This audit included all cases discussed at departmental discrepancy meetings between April 2022 and March 2024. Minutes were reviewed, and cases were included if a confirmed diagnostic discrepancy was identified. Variables collected included specimen type, specialty, detection method, category of discrepancy, stage of error (pre-analytical, analytical, post-analytical), root cause and clinical impact (see Appendix, Table 3).

Descriptive statistics were used. Counts (N) and percentages were presented together. No inferential statistics (p-values, χ², t-tests) were performed, as this was an observational audit rather than a comparative study.

Discrepancy category definitions

Category A: Macroscopic discrepancies (sampling, labelling, grossing errors); Category B: Microscopic interpretation discrepancies: B1-An error in an obvious diagnosis, B2-A moderately difficult diagnosis, B3-A very difficult diagnosis; Category C: Lack of clinical correlation; Category D: Failure to seek a second opinion; Category E: Reporting/transcription errors.

## Results

Six of the 32 cases were excluded from the analysis because it was decided there was no discrepancy after discussion. A total of 38,824 specimens were processed during the study period. The overall number of cases with discrepancies identified was 26, with an average of one case per month and an error rate of 0.06%, which is highlighted in Table 1.

**Table 1:** Number of discrepancies in years 2022 and 2023.

Year	Number of discrepancies
2022	5
2023	19

Those discrepancies were distributed as follows: 

Discrepancy Category

Two of the cases had two different types of errors, so the total number of errors in this part of the analysis is 28. Following the Royal College of Pathologists' guidelines on the classification of diagnostic discrepancies identified through expression of concern about a doctor's performance: 16 cases (56%) of discrepancies fell in category B (discrepancy in microscopy). Most of which were either very difficult diagnoses or cases with large interobserver variation.

The second most frequent category was A (macroscopic discrepancy), where six cases (21%) were due to inadequate sampling, and one of those cases was also mistakenly labelled. Three cases (11%) were due to failure to seek a second opinion in an obviously difficult case (category D). 

**Figure 1:**
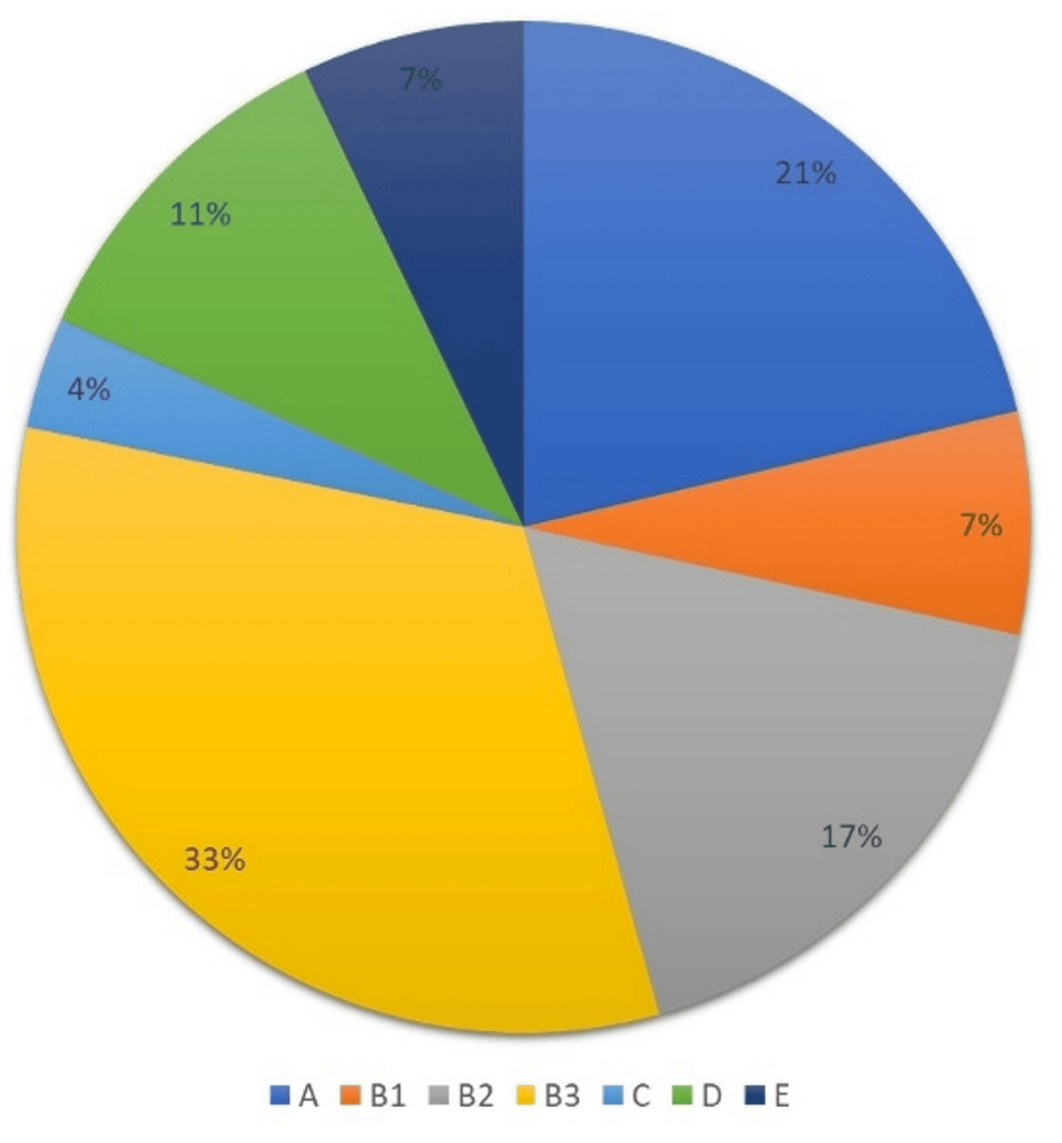
Types of discrepancies.

Less common discrepancy categories were the lack of clinical correlation (category C), where one case was identified (3.5%). Two cases (7%) were due to a discrepancy in reporting (category E). This is highlighted in Table 2 and Figure 1.

**Table 2:** Discrepancy categories.

Discrepancy category	Number of cases (N)	Percentage (%)
A	6	21%
B1	2	7%
B2	5	17%
B3	9	32%
C	1	3.5%
D	3	11%
E	2	7%

The Number and Nature of Cases 

**Figure 2:**
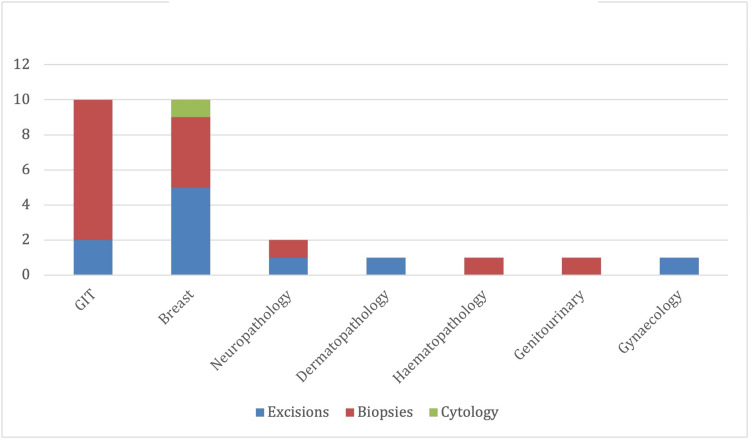
Subspecialty and sample type. GIT: gastrointestinal tract.

The specialties discussed included: breast pathology, gastrointestinal tract (GIT) pathology, neuropathology, dermatopathology, respiratory pathology, hematopathology, gynecological pathology, and genitourinary pathology (Figure 2). There were 15 (53.5%) biopsies, 10 resections (35%), and one cytology (3.5%) specimen.

The majority of diagnostic inaccuracies (16) were among biopsy cases (breast (four)-GIT (eight)). Vacuum-assisted biopsies (VAB) accounted for four (40%) of all breast discrepancies. The most frequent discrepancies were in GIT (10) and breast (10) specimens. Fewer discrepancies occurred in neuropathology (two), haematology (one), dermatopathology (one), genitourinary (one), and gynaecology (one).

*Effect on Patient Care* 

The overall incidence of errors with patient consequences was 0%. All cases were reviewed and amended without patient harm or delay in patient care. 

Case Identification 

**Figure 3:**
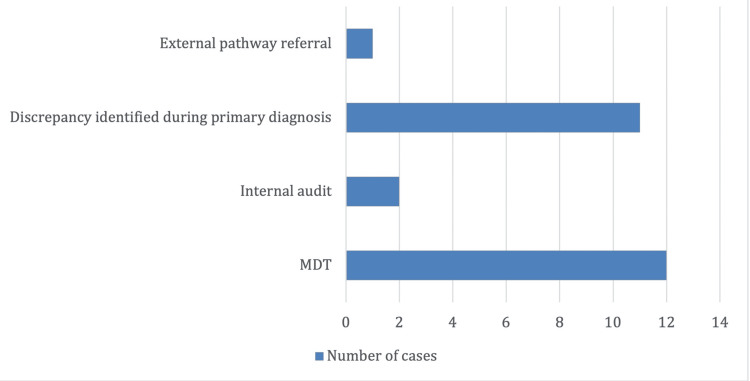
Case identification. MDT: multidisciplinary team.

The cases were identified either by multidisciplinary team (MDT) review 12 (46.1%), internal audits 2 (7.7%), or identified during primary diagnosis due to suspicion or correlation to clinical data or a related specimen 11 (42.3%). One discrepancy was identified through external pathway referrals. The method of identification is highlighted in Figure 3.

Stage of Workflow Where the Error Stemmed 

The pre-analytical phase encompassed all specimen handling activities prior to the pathologist's review of the slide (n = 6, 21.4% of cases). The analytical phase referred to diagnostic mistakes occurring during microscopic examination (n = 20, 71.4% of cases). The post-analytical phase pertained to errors arising in the preparation of the diagnostic pathology report (n = 2, 7.1% of cases).

## Discussion

The primary objective of this study was to investigate the discrepancies at PAWS cellular pathology during all three phases of pathology workup: pre-analytical, analytical, and post-analytical.

The calculated overall error rate was 0.06%. Although no concrete standard was identified by the Royal College of Pathologists as an acceptable error rate, we tried to benchmark against published studies dealing with discrepancies at the histopathology lab. By comparison, according to a published study, the rate of inaccurate diagnoses (assessed as a major discordance) ranged from 3% to 9% among the different specimen groups, with the highest mean percentage of inaccurate diagnoses being in gynaecology, dermatopathology, and gastrointestinal specimens ​[3]​. In a USA study in 2006, the acceptable error rate was determined to be between 0.5 and 1% ​[4]​. Based on this, it appears that the error rate at our department appears to be within acceptable limits.

The patterns of error identified in our results are diverse, encompassing a wide range of organ systems and specimen types. Although numerous interpretative tendencies were found to be prone to error, this discussion focuses on three of the most common patterns, illustrated with specific case examples. The first involves the number of levels taken in vacuum-assisted biopsy (VAB) samples in breast pathology, which can significantly influence diagnostic accuracy. The second relates to suboptimal sampling of mastectomy specimens, particularly in cases where no palpable lesions are easily identified, potentially leading to incomplete assessment. The third concerns difficult or subjective cases that require pre-multidisciplinary team (MDT) discussions, where reaching a consensus diagnosis can be challenging due to interpretative variability among pathologists. 

Below, we discuss all these issues with further details. A problem recurred with VAB cases, which is the difficulty of reaching an agreement around the number of levels that should be initially cut from the block. 

The current Royal College of Pathologists' dataset for histological reporting of breast cancer states that, for core biopsies of mass lesions, a single haematoxylin and eosin-stained section is generally adequate. However, when core biopsies are performed to evaluate microcalcifications, at least three levels should be assessed. In challenging cases, examining additional levels and employing immunohistochemical techniques can provide further diagnostic support [5].

We had a recurring issue in most of the cohorts we analysed of breast cases sampled for microcalcifications, where at least three levels were initially examined and reported as benign. However, upon review, further levels were requested (based on clinical query, or a concerning focus being picked up on MDT review, or while reporting another related specimen), and in those, atypia or malignancy were identified. This highlighted the importance of a Faxitron (Hologic, Inc., Marlborough, MA, USA) as a solution for this recurring scenario, since it can pick up the calcifications and highlight the most densely calcified areas in the tissue block. This can potentially allow targeted sampling and sectioning of the specimen and tissue block, respectively.

Additionally, Faxitron can help detect calcifications or biopsy marker clips in cases of ductal carcinoma in situ (DCIS) in mastectomies or post-adjuvant treatment samples. In a 2012 study, the use of Faxitron was proven to be more effective in identifying microcalcifications and assessing surgical margins ​[6]​. Another study also showed that in-lab imaging of breast cases did not increase turnaround time, unlike having to seek help from the mammography suite imaging. They also required fewer blocks compared to specimens that were not scanned in-lab ​[7]​. 

Thus, based on the discussion in the meeting, we as a department found it imperative to acquire a Faxitron, which could potentially alleviate the recurrent problems that were brought to the discussion in VAB specimens and mastectomies with no palpable lesions.

In this audit, 46.1% of discrepancies were identified through MDT meetings. While some departments are adopting the practice of review of paper reports rather than slides to save time in the face of increasing workload, based on our data, the most robust way of uncovering discrepancies was MDT slide review ​[8]​. This was also highlighted by a study by O'Connor and colleagues, where it was found that MDT review made it more likely to detect an error; however, it adds a significant workload for the participating pathologists ​[9].

In one study of multidisciplinary team meetings at a teaching hospital in Ireland, the number of meetings attended by the histopathology department ranged from two to eight per day, excluding grand rounds, amounting to approximately 50 meetings per month. Over one month, pathologists spent over 300 hours at 81 meetings. 2.4 histopathology hours were spent in preparation for each meeting [10].​

Several cases also highlighted the need for a consensus meeting, particularly in upper GIT samples, where there is a disagreement between the primary reporting pathologist and the reviewing pathologist during MDT review, or when a case is difficult and subjective, and more than one opinion needs to be sought. There is usually a very limited time for case review prior to the MDT. In many instances, a definitive opinion would require patient discussion to be postponed to the following week, until the next MDT, as it can be difficult to give a definitive opinion to the clinical team during the MDT if a disagreement arises. 

In a study by Arif and Wong, the importance of consensus/pre-MDT review was highlighted as it served multiple purposes, ensuring no internal inconsistencies, confirming the primary diagnosis, and identifying areas for refinement. It also serves as an educational function for participating pathologists and serves a quality assurance role ​[11]​. 

It is also worth noting that during the study period, agency locum staff were employed by the department to help resolve workload pressures. A significant percentage of the cases listed for discussion were reported by the agency staff, although no exact figure was produced for that in our audit to maintain anonymity. When analysing a common theme for why the errors happened, it appears that it boils down to either not having adequate discussions with colleagues or not doing the necessary further work. This is likely due to the working pattern of an agency locum, where there is probably a need to complete the work as quickly as possible, due to the nature of the hourly remuneration contract [12].

Based on this, we glean that MDT slide reviews are of paramount importance to pick up possible discrepancies. The high workload and limited time have cast some limitations on our practice, hindering the formulation of a pre-MDT/peer review discussion system, which we have detailed the importance of, and the potential added value. Additionally, the overstretched departments can resort to agency locums to fill the staffing gaps, as we did for a while at Salford, which comes at a cost to quality and accuracy. 

Factoring everything in, the histopathology practice at Salford Royal remains a high-quality service, with acceptable error rates.

Recommendations

We should allocate protected time for MDT preparation and participation within job plans. In addition to this, we should procure Faxitron for improved breast biopsy accuracy. Teams should have a consensus for cases with interobserver variability. Lastly, we should restrict agency locum work to non-urgent or routine cases to maintain quality. 

## Conclusions

The diagnostic discrepancy rate in the PAWS cellular pathology department was low and within acceptable limits compared with published data. No discrepancies caused patient harm, demonstrating effective internal safety mechanisms. MDT slide review was the most sensitive method of error detection. The audit highlights the need for improved sampling of breast VAB specimens and protected pre-MDT preparation time. Strengthening peer-review structures and reducing reliance on short-term locum staff may improve diagnostic consistency. Regular discrepancy review remains essential for education and quality improvement.

## Appendices

**Table 3:** Data collection pro forma. Datix (y/n): Indicates whether a formal Datix incident report was submitted. Datix is the hospital's electronic incident-reporting system used for documenting safety events and governance follow‑up.

Case number				
Date				
Subspecialty				
Sample type				
Specimen				
History				
Initial diagnosis				
Method of identification				
Changes implemented				
Final diagnosis				
Discrepancy category				
Outcome				
Action taken				
Datix (y/n)				
